# Anti-obesity effects of *Lactiplantibacillus plantarum* SKO-001 in high-fat diet-induced obese mice

**DOI:** 10.1007/s00394-023-03096-x

**Published:** 2023-02-02

**Authors:** Mi Jin Choi, Hana Yu, Jea Il Kim, Hee Seo, Ju Gyeong Kim, Seul-Ki Kim, Hak Sung Lee, Hyae Gyeong Cheon

**Affiliations:** 1 Department of Pharmacology, Gachon University School of Medicine, Incheon, 21999 Republic of Korea; 2 Department of Health Sciences and Technology, GAIHST, Incheon, 21999 Republic of Korea; 3 Food Science R&D Center, Kolmar BNH CO., LTD, 61, Heolleung-ro 8-gil, Seocho-gu, Seoul, 06800 Republic of Korea

**Keywords:** Anti-obesity, Probiotics, Lipogenesis, Fibrosis, Browning, High-fat diet

## Abstract

**Purpose:**

Previous reports showed that some probiotics provide beneficial effects on various diseases including metabolic disorders. This study aimed to investigate the anti-obesity effects of *Lactiplantibacillus* (*L.*) *plantarum* SKO-001 (SKO-001), a probiotic strain newly isolated from *Angelica gigas*.

**Methods:**

C57BL/6J mice were fed with high-fat diet (HFD, 60% fat) for four weeks, and then different doses of SKO-001 (*n* = 10 each group) were orally given for 12 weeks. Following treatment, body weight, fat weight, serum parameters and adipose and liver tissues were analyzed.

**Results:**

SKO-001 (2 × 10^10^ CFU/day, *per os*) reduced body weight gain after 10th week of administration, accompanied by a reduction in body fat mass of mice. In the SKO-001-fed group, increased serum adiponectin, decreased leptin, insulin, total cholesterol, low-density lipoprotein cholesterol, free fatty acids, and triglyceride levels were observed. Hematoxylin and eosin staining of various fat depots showed that increased adipocyte size caused by HFD intake was markedly reduced and correlated with reduced mRNA levels of lipogenesis genes, including sterol regulatory element-binding protein-1c, peroxisome proliferator-activated receptor gamma, and CCAAT/enhancer binding protein alpha, and increased uncoupling protein 1 levels. Similarly, SKO-001 reduced lipid accumulation, decreased the mRNA levels of lipogenic genes, and reduced α-smooth muscle actin and collagen type 1 alpha 1 levels in the liver.

**Conclusions:**

SKO-001 ameliorates obesity and related metabolic abnormalities in adipose and liver tissues, possibly via the regulation of lipid metabolism. Based on the results of the present study, SKO-001 may be applicable as an anti-obesity therapeutic or functional food.

**Supplementary Information:**

The online version contains supplementary material available at 10.1007/s00394-023-03096-x.

## Introduction

Obesity is the most important risk factor for metabolic disorders, including insulin resistance, type 2 diabetes mellitus, dyslipidemia, non-alcoholic fatty liver disease, and cardiovascular disease [1, 2]. Many factors, including unhealthy diet, genetic background, and sedentary lifestyle, lead to the development of obesity. Recently, alterations in the intestinal microbiota resulting from unhealthy diet have been shown to contribute to the pathogenesis of obesity [3]. Apart from bariatric surgery [4], several pharmacological agents are available for the treatment of obesity, among which appetite suppressants are popularly prescribed despite their central nervous system-related side effects [5].

The most prominent characteristic of obesity is excessive lipid accumulation in the body, resulting from an imbalance between lipogenesis and lipolysis under certain conditions [6]. The major tissues responsible for obesity are the adipose tissues, which are not mere depots for fat storage, but also act as an active endocrine organ and secrete various adipokines, including adiponectin, leptin, and resistin [7, 8]. Upon obesity development, adipocytes become enlarged, consequently provoking impaired insulin sensitivity and proinflammatory and diabetogenic conditions [9]. Interestingly, browning, the conversion of white adipocytes (enlarged spherical cells storing lipids) to brown-like adipocytes (small cells dissipating heat), reduces insulin resistance and obesity, and induction of uncoupling protein-1 (UCP-1) is a distinct marker of browning [10–12].

Chronic obesity results in a failure to manage the increased energy influx, leading to the storage of ectopic fat in tissues, including the liver and muscles. Abnormal hepatic lipid accumulation promotes non-alcoholic fatty liver disease, leading to liver fibrosis and cirrhosis [13]. The pathogenesis of hepatic fibrosis appears to be complex, where lipotoxicity caused by toxic lipid metabolites drives the progression of hepatic fibrosis via activation of hepatic stellate cells [14]. During liver fibrosis, activated hepatic stellate cells increase the levels of α-smooth muscle actin (α-SMA), remodeling the extracellular matrix by secreting matrix metalloproteases and depositing components, including collagen type 1 alpha 1 (Col1α1) [15]. Several mediators are involved in lipid metabolism, including sterol regulatory element-binding protein-1c (SREBP-1c) and CCAAT/enhancer binding protein alpha (C/EBPα), which are important transcription factors involved in the regulation of lipogenic gene mRNA expression. In addition, peroxisome proliferator-activated receptor gamma (PPARγ) and C/EBPα are key regulators of adipogenesis [16, 17].

Much effort has been focused on discovering natural products as supplementary or alternative anti-obesity agents, since these materials have long been used for treating various diseases without noticeable toxicity. Some probiotics confer healthy conditions to the host [18] by normalizing the impaired lipid and glucose metabolism, altering the composition of gut microbiota, and suppressing metabolic inflammation [19, 20]. For example, *Lactiplantibacillus* *plantarum* K21 for eight weeks of administration significantly reduced body weight gain, epididymal fat mass accumulation, and serum leptin levels in a high-fat diet (HFD)-induced obese mouse model [21]. Similarly, eight weeks of administration of *L. plantarum* LMT1-48 to HFD obese mice reduced body weight and abdominal fat volume concurrently with downregulation of lipogenic genes [22]. Reduction in body weight and body mass index by *L. plantarum* Dad-13 intake for 12 weeks has been reported [23]. Although the exact mechanisms and effects of probiotics are strain- and dose-dependent, several studies have demonstrated that probiotic intervention can be a safe treatment strategy for mitigating obesity [24–26]. Thus, in the present study, we investigated the anti-obesity effects of newly isolated *L. plantarum* SKO-001 (SKO-001) in HFD-induced obese mice.

## Materials and methods

### Reagents

Oligonucleotide primers specific for glyceraldehyde 3-phosphate dehydrogenase (*GAPDH*), *SREBP-1c*, *C/EBPα, PPARγ, UCP-1, αSMA,* and *Col1α1* were purchased from Bioneer (Daejeon, Korea). M-MLV reverse transcriptase and random oligonucleotide primers were from Promega (Fitchburg, WI, USA). TOP script RT Dry MIX was from Enzynomics Co., Ltd. (Daejeon, Korea). Thunderbird SYBR quantitative polymerase chain reaction (qPCR) Mix was from TOYOBO Co., Ltd. (Osaka, Japan). All other chemicals were from Sigma-Aldrich Chemical Co. (St. Louis, MO, USA).

### Preparation of SKO-001

SKO-001 (Accession No. KCTC 14816BP) was isolated from *Angelica gigas* Nakai and prepared by Kolmar BNH Co., Ltd. (Seoul, Korea). Briefly, SKO-001 was anaerobically incubated in the culture medium (glucose, yeast extract, peptone, polysorbate 80, calcium chloride, magnesium sulfate, manganese (II) sulfate monohydrate, and LS-300) at 37 ± 2 ℃ for 16 h. After incubation, the cells were harvested via centrifugation at 9000×*g*, lyophilized, and stored at 4 ℃ until use. Freeze-dried SKO-001 powder was freshly suspended in autoclaved tap water daily for administration to animals.

### In vivo studies

Six-week-old male C57BL/6J mice were purchased from Orient Bio (Seoul, Korea). The animals were acclimatized under the following conditions for seven days: temperature, 23 ± 2 ℃; humidity, 40–60%; circadian cycle, 12-h light/dark cycle. The mice were divided into the following five groups (*n* = 10/group): (1) control group, fed a normal chow diet (ND) and treated with vehicle (autoclaved tap water); (2) vehicle group, fed HFD (60% fat) for four weeks, followed by treatment with vehicle; (3) SKO-001-L group, fed HFD, followed by treatment with SKO-001 (5 × 10^9^ CFU/day); (4) SKO-001-M group, fed HFD, followed by treatment with SKO-001 (1 × 10^10^ CFU/day); and (5) SKO-001-H group, fed HFD, followed by treatment with SKO-001 (2 × 10^10^ CFU/day). During the treatment period, body weight, food intake, and blood glucose levels were measured weekly at the same time of the day (between 10:00 and 11:00 AM). The amount difference between the supplied food and remaining food was considered as food intake. Blood was obtained by tail vein puncture, and analyzed glucose levels using Allmedicus GlucoDr Plus (Seoul, Korea). After 12 weeks of treatment, the animals were subjected to the assessment of fat and lean body mass using the ^1^H minispec system (Bruker BioSpin; Billerica, MA, USA). After overnight fasting, the animals were euthanized with isoflurane, and blood samples were collected via cardiac puncture and centrifuged at 3000×*g* for 15 min. Adipose and liver tissues from different depots were carefully excised and weighed. Part of the tissues were frozen in liquid nitrogen and stored at – 80 °C until further analysis. The other part of the adipose tissue was collected, fixed with 4% paraformaldehyde, and sectioned for UCP-1 immunostaining and hematoxylin and eosin (H&E) staining. Referring to the location of fat isolated from the mice, following regions were isolated; subcutaneous white adipose tissue (SAT) from the inguinal region; epididymal white adipose tissue (EAT) from the region located in the lower part of the abdomen and connected to the epididymis; visceral white adipose tissue (VAT) from the areas surrounding the intestine. All animal procedures were carried out in accordance with the Guide for the Care and Use of Laboratory Animals published by the US National Institutes of Health (NIH Publication No. 85–23, revised 2011) and were approved by the Animal Care and Use Committee of Gachon University, South Korea (approval No. LCDI-2021–0059).

### RNA preparation and reverse-transcription-qPCR (RT-qPCR)

The isolated RNA from adipose and liver tissues was reverse-transcribed into complementary DNA using the TOP script RT Dry MIX and Oligo dT primers (Promega). RT-qPCR analysis was performed in triplicates for each sample. The expression of target genes was normalized to that of *GAPDH*. PCR conditions were as follows: 95 ℃ for 10 min (initial denaturation), followed by 45 cycles of 95 ℃ for 15 s (denaturation), 53 ℃ for 1 min (annealing), and 72 ℃ for 30 s (elongation). The sequences of the primer sets used in this study are listed in Table 1.

**Table 1:** Primer sequences used for reverse transcription-quantitative polymerase chain reaction (RT-qPCR)

Gene	Forward (5′–3′)	Reverse (3′–5′)
*UCP-1*	CAAAAACAGAAGGATTGCCGAAA	TCTTGGACTGAGTCGTAGAGG
*SREBP-1c*	CGACTACATCCGCTTCTTGCAG	CCTCCATAGACACATCTGTGCC
*PPARγ*	GTACTGTCGGTTTCAGAAGTGCC	ATCTCCGCCAACAGCTTCTCCT CCTTCT
*C/EBPα*	GCAAAGCCAAGAAGTCGGTGGA	GTTGCGTCTCCACGTT
*αSMA*	TGCTGACAGAGGCA CCACTGAA	CAGTTGTACGTCCAGAGGCATAG
*Col1α1*	CCTCAGGGTATTGCTGGACAAC	CAGAAGGACCTTGTTTGCCAGG
*GAPDH*	GTCTCCTCTGACTTCAACAGCG	ACCACCCTGTTGCTGTAGCCAA

### H&E staining and UCP-1 immunostaining of adipose tissues

Paraffin-embedded tissue Sects. (4–6 μm) were deparaffinized, rehydrated, permeabilized with 0.1% Triton X-100 (1 h, room temperature), and washed with phosphate-buffered saline (PBS). The tissue sections were stained with H&E, and images were captured using an Axio Imager Z1 microscope (Carl Zeiss Microscopy, Oberkochen, Germany). For UCP-1 immunostaining, the sections were incubated with primary polyclonal antibodies against UCP-1 (Abcam, Cambridge, UK; diluted 1:200) at 4 °C overnight. After washing with PBS, the sections were incubated with Alexa Fluor 488 (Invitrogen, Waltham, MA, USA; diluted 1:300). After washing with wash buffer, the sections were mounted with VECTASHIELD mounting medium containing 2-(4-amidinophenyl)-6-indolecarbamidine dihydrochloride (DAPI; Vector Laboratories, Newark, CA, USA) and examined under a Zeiss LSM700 confocal microscope (Carl Zeiss Microscopy). Using the Viewpoint-Viewer software (PreciPoint, Freising, Germany), the size of adipocytes in the field was determined.

### α-SMA immunostaining of liver tissues

Liver tissues fixed in 10% neutral buffered formalin were embedded in paraffin after washing with PBS. The Sects. (4–6 μm) were incubated with primary polyclonal antibodies against α-SMA (Novus Biologicals, Littleton, CO, USA; diluted 1:200) at 4 °C overnight. After washing with PBS, sections were incubated with Alexa Fluor 488 (Invitrogen; diluted 1:300). After washing with wash buffer, the sections were mounted with VECTASHIELD mounting medium containing DAPI and examined using a Zeiss LSM700 confocal microscope.

### Oil Red O staining

Liver tissues were fixed in 10% neutral buffered formalin for frozen sectioning (– 15 °C). Frozen Sects. (20-µm thickness) were incubated with 60% isopropanol  for 5 min and stained with 0.3% Oil Red O/60% isopropanol solution for 10 min after complete drying, followed by five dips of alum hematoxylin. After rinsing with distilled water, images of the lipid droplets were visualized and captured under an Axio Imager Z1 microscope.

### Analysis of serum biochemical markers

Serum levels of various markers were determined using commercial kits according to the protocols provided by each vendor: free fatty acid (FFA; Abcam), low-density lipoprotein cholesterol (LDL-C)/high-density lipoprotein cholesterol (HDL-C; Abcam), insulin (ALPCO, Macedon, NY, USA), adiponectin (ALPCO), triglyceride (Abbkine Scientific Co. Ltd., CA, USA), total cholesterol (TC; Abbkine), FFA (Abbkine), and leptin (Abbkine).

### Statistical analysis

The results are expressed as the mean ± standard deviation for each group. The means between the treatment groups were compared using Student’s *t*-test or two-way analysis of variance. Statistical significance between groups was considered at *p* < 0.05.

## Results

### SKO-001 suppresses HFD-induced increase in body and fat weights

**Fig. 1:**
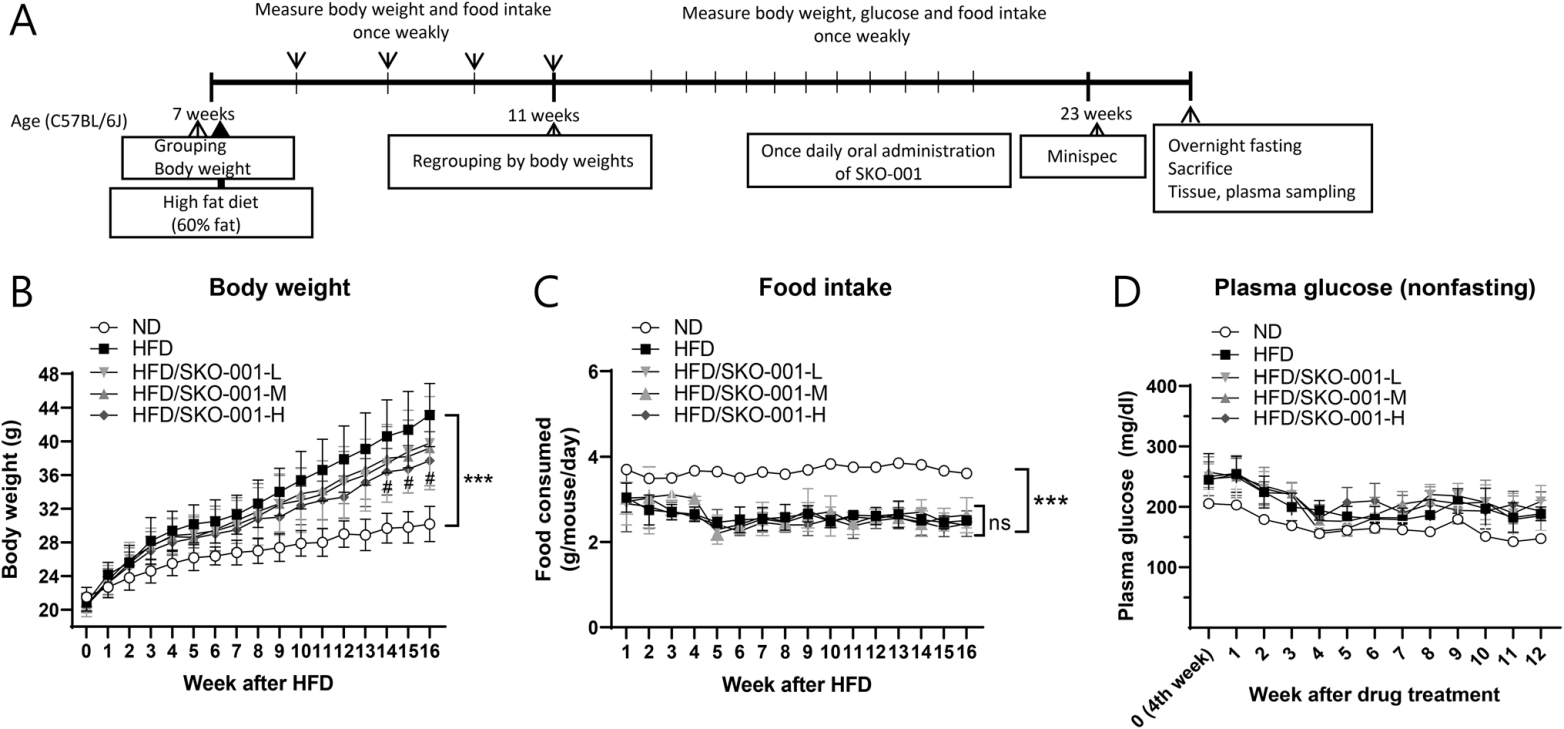
Effects of SKO-001 on body weight, food intake, and plasma glucose levels in HFD-induced obese mice. C57BL/6J mice (seven weeks old, male) were fed either a normal chow diet (ND) or HFD for four weeks. HFD-induced obese mice were orally administered SKO-001 (three different doses; once daily) for 12 weeks. Experimental scheme is shown (**A**). Body weight (**B**), food intake (**C**), and plasma glucose levels (**D**) were measured at the indicated days **(***n* = 10/group**)**. Values are represented as the mean ± standard deviation (SD). *ns* not significant. ***p < 0.001 *vs.* ND condition. ^#^*p* < 0.05 *vs*. HFD condition

**Fig. 2:**
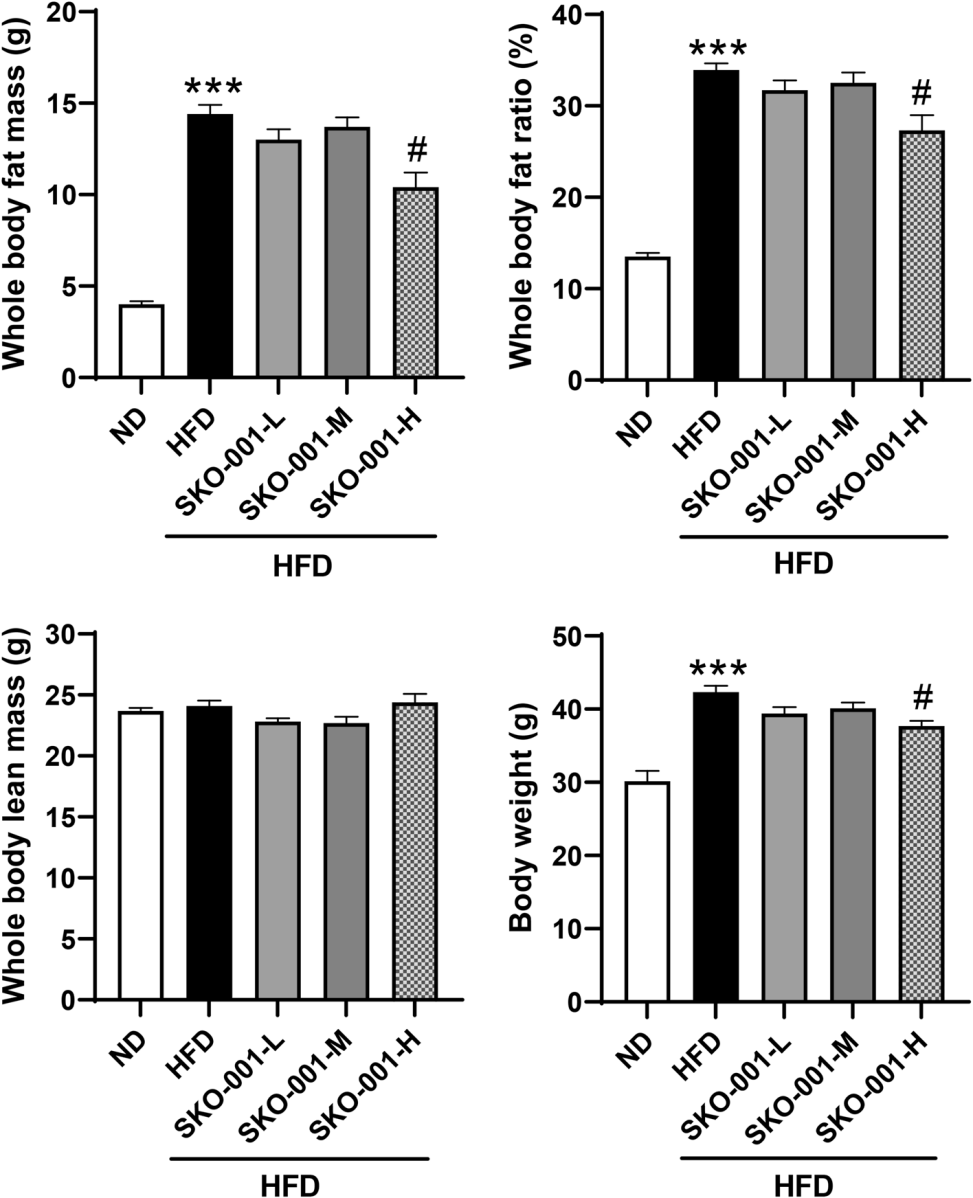
Effect of SKO-001 on fat mass in HFD-induced obese mice. After 12 weeks treatment of mice with SKO-001, the animals were subjected to fat mass measurements. Fat and lean body mass were assessed using the ^1^H minispec system. Values are represented as the mean ± SD. ***p < 0.001 *vs.* ND condition. ^#^*p* < 0.05 *vs*. HFD condition

To determine the anti-obesity effects of SKO-001, C57BL/6J mice were fed a HFD (60% fat) for four weeks, followed by oral administration of different doses of SKO-001 (SKO-001-L, 5 × 10^9^ CFU/day; SKO-001-M, 1 × 10^10^ CFU/day; SKO-001-H, 2 × 10^10^ CFU/day) once daily for 12 weeks (Fig. 1A). As shown in Fig. 1B, HFD feeding induced significant body weight gain throughout the experimental period compared with ND feeding. Upon oral administration of SKO-001, body weight gain was reduced starting from 10th week administration (SKO-001-H). At the end of the experiments, the vehicle group (HFD group) gained 43.1 ± 3.76 g, while SKO-001-H-fed group gained 37.7 ± 3.45 g body weight (12.7% decrease vs. HFD group; *p* < 0.05). Meanwhile, little alteration in feed intake and non-fasting glucose levels were observed in the SKO-001-treated groups, although feed intake between the ND- and HFD-fed groups was considerably different (Fig. 1C, D). Consistent with the effects of SKO-001 on body weight, the HFD-induced increase in body fat mass measured by minispec was also significantly suppressed in the SKO-001-H group without any change in whole-body lean mass (Fig. 2), suggesting that decreased body weight gain by SKO-001 may be attributed to the selective reduction of fat mass.

### SKO-001 improves blood parameters in HFD-induced obese mice

**Fig. 3:**
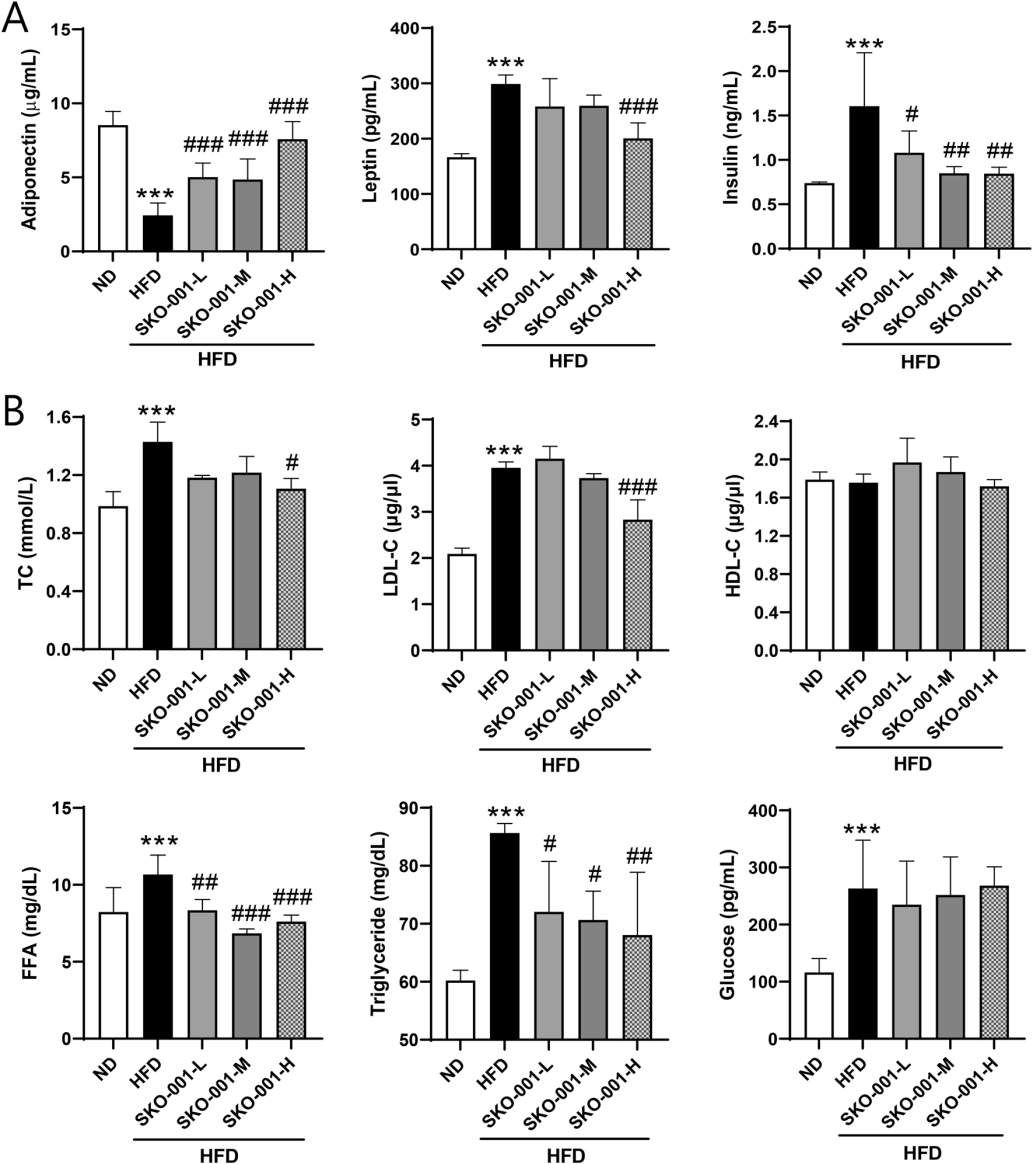
Effects of SKO-001 on serum biochemical parameters in HFD-induced obese mice. HFD-induced obese mice were orally administered SKO-001 for 12 weeks. After fat mass measurement, the mice were subjected to overnight fasting, and blood samples from each mouse were taken via cardiac puncture the next morning. Various serum parameters were determined using enzyme-linked immunosorbent assay (ELISA) kits as indicated in the Materials and methods section. **A** Adiponectin, leptin, and insulin levels. **B** Total cholesterol (TC), low-density lipoprotein cholesterol (LDL-C), high-density lipoprotein cholesterol (HDL-C), free fatty acid (FFA), triglyceride, and glucose levels. Values are represented as the mean ± SD. ***p < 0.001 *vs.* ND condition. ^#^*p* < 0.05, ^##^*p* < 0.01, ^###^*p* < 0.001 *vs*. HFD condition

Reduced body and fat weights are associated with increased whole-body insulin sensitivity. Thus, the plasma levels of various cytokines were determined after 12 weeks of oral administration of SKO-001 to examine its effects on insulin sensitivity. Two important cytokines related to insulin sensitivity, namely, adiponectin and leptin, were altered by HFD feeding: increased leptin and reduced adiponectin levels (Fig. 3A). The reduced levels of adiponectin induced by HFD feeding were recovered in a dose-dependent manner by SKO-001. Conversely, the HFD-induced leptin levels were normalized to levels similar to those in the ND group after SKO-001 treatment (Fig. 3A). While SKO-001 had little effect on plasma glucose levels compared with the HFD group, plasma insulin levels were markedly reduced by SKO-001 treatment (Fig. 3A), suggesting that SKO-001 may improve insulin resistance developed by chronic HFD feeding. Moreover, overall lipid profiles were ameliorated in SKO-001-treated groups, that is, the levels of TC, LDL-C, FFA, and triglycerides decreased markedly in SKO-001-treated groups than in the HFD group (Fig. 3B), suggesting that SKO-001 is effective for recovering abnormal lipid metabolism induced by HFD feeding.

### SKO-001 ameliorates lipid metabolism-related parameters in adipose tissues

**Fig. 4:**
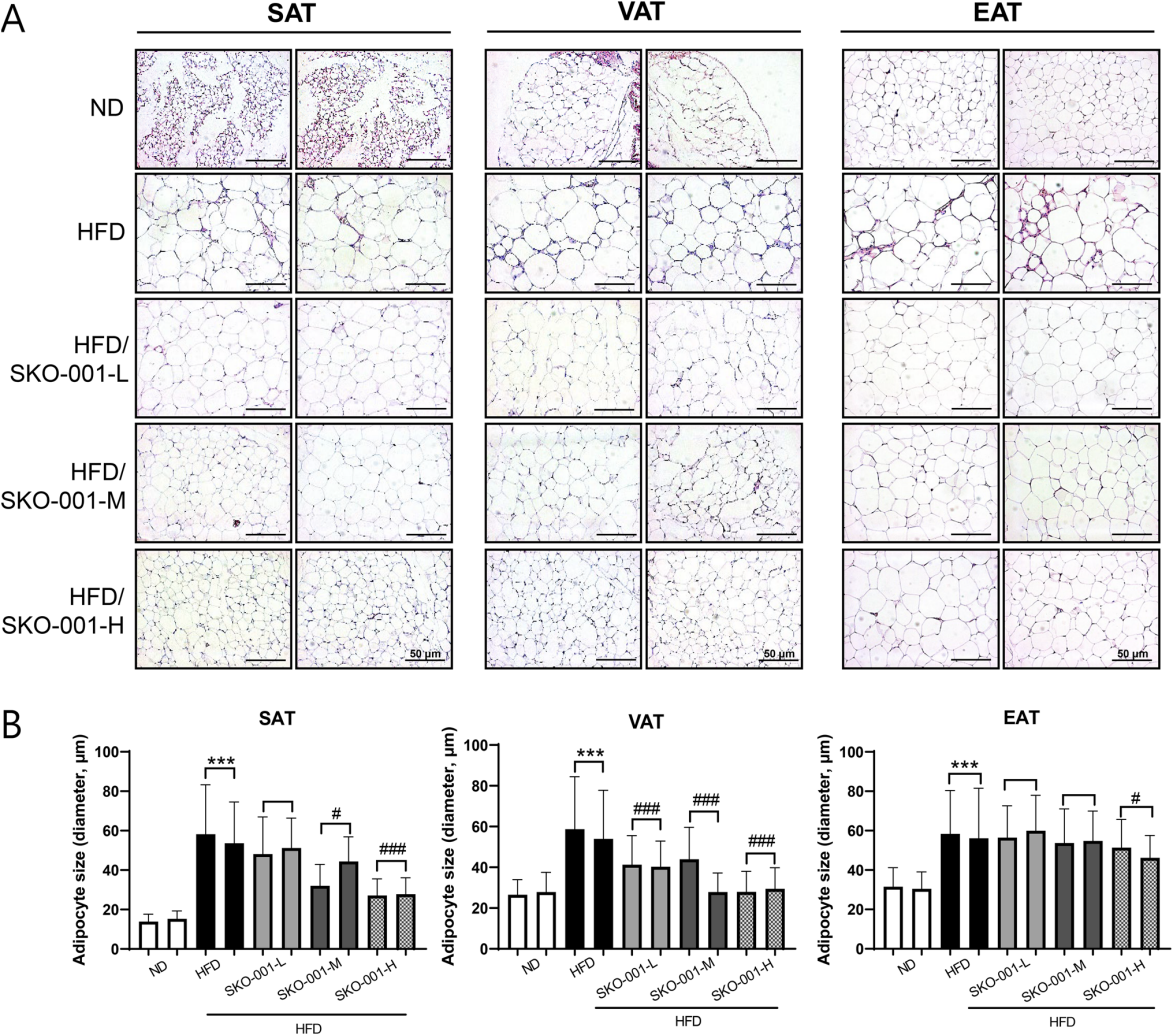
Effects of SKO-001 on adipocyte size in the adipose tissues of HFD-induced obese mice. After cardiac puncture to obtain blood samples from each group, the adipose tissues were isolated, and paraffin-embedded tissue sections were stained with hematoxylin and eosin (H&E). Images were captured under the microscope, Axio imager Z1 (**A**) (Scale bar = 50 μm, 200 × magnification). The cell size in each group was estimated using Viewpoint-viewer software program (**B**). SAT; subcutaneous white adipose tissue, VAT; visceral white adipose tissues, EAT; epididymal white adipose tissue. Values are represented as the mean ± SD. ***p < 0.001 *vs.* ND condition. ^#^*p* < 0.05, ^###^*p* < 0.001 *vs*. HFD condition

**Fig. 5:**
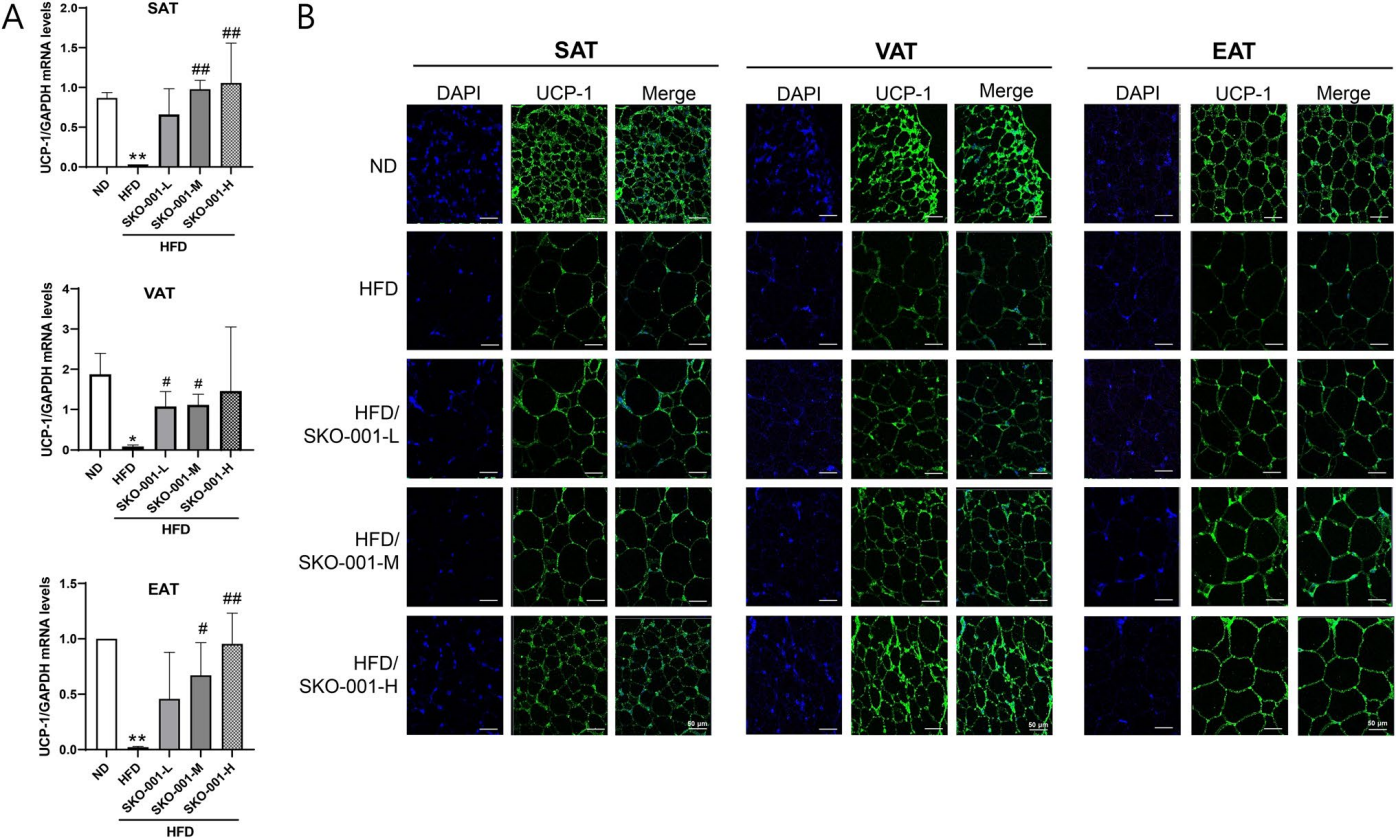
Effects of SKO-001 on UCP-1 expression in the adipose tissues of HFD-induced obese mice. Adipose tissues isolated from each group were subjected to RT-qPCR analysis. The mRNA levels of *UCP-1* were determined using real-time RT-qPCR (repeated three times, each in triplicate; **A**. UCP-1 immunostaining was carried out using paraffin-embedded tissue sections (**B**; Scale bar = 50 μm, 200 × magnification). Values are represented as the mean ± SD. *p < 0.05, **p < 0.01 *vs.* ND condition. ^#^*p* < 0.05, ^##^*p* < 0.01 *vs*. HFD condition

**Fig. 6:**
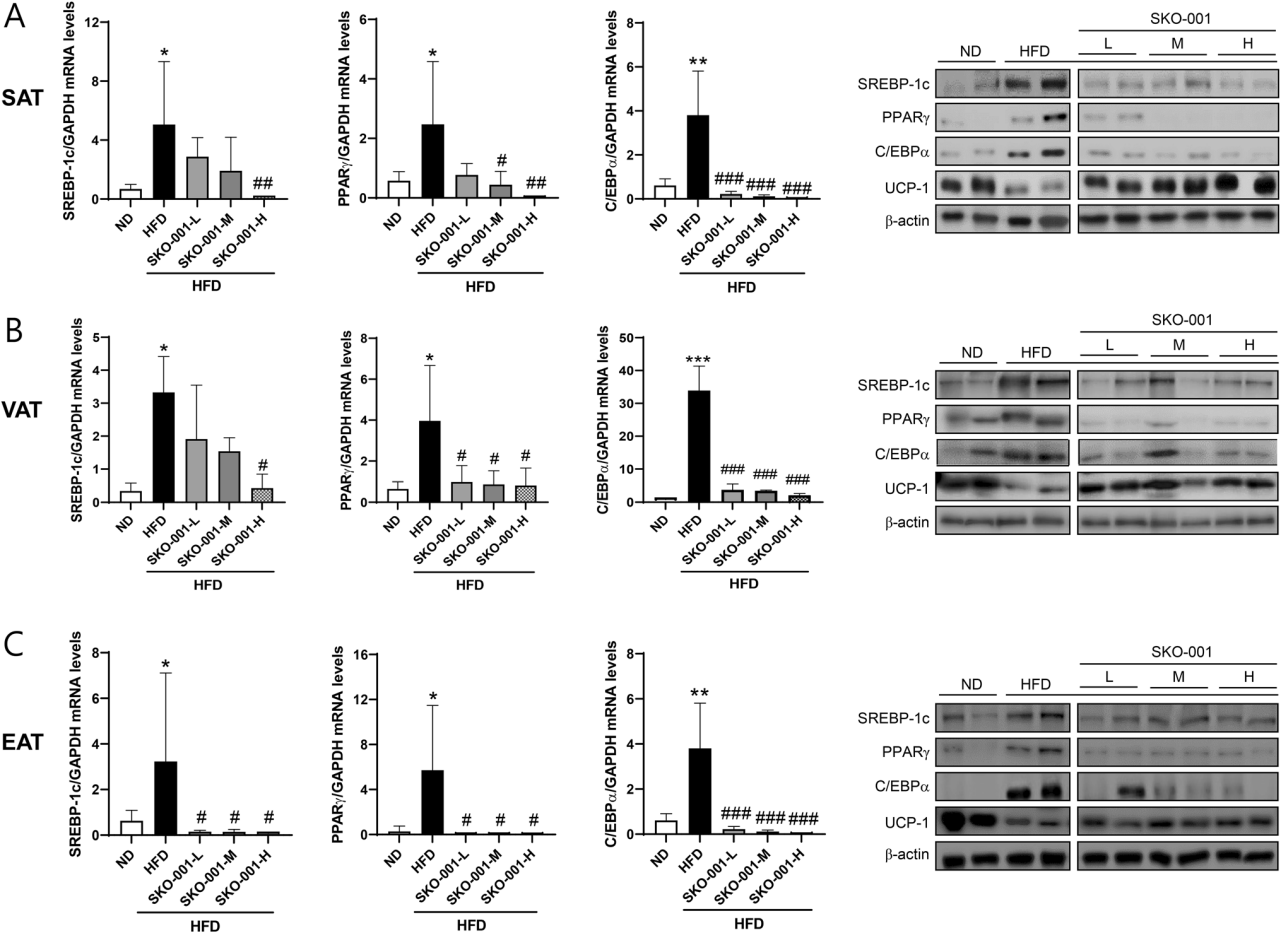
Effects of SKO-001 on the mRNA expression of genes involved in lipogenesis in the adipose tissues of HFD-induced obese mice. Adipose tissues isolated from each group were subjected to RT-qPCR analysis. The mRNA levels of lipogenesis markers (*SREBP-1c*, *PPARγ*, and *C/EBPα*) were determined via real-time RT-qPCR (repeated three times, each in triplicate; **A**–**C** left panel). Protein levels were determined via western blotting analysis (**A**–**C** right panel). Values are represented as the mean ± SD. *p < 0.05, **p < 0.01 *vs.* ND condition. ^#^*p* < 0.05, ^##^*p* < 0.01, ^###^*p* < 0.001 *vs*. HFD condition

Analysis of metric and biochemical parameters revealed that SKO-001 improved HFD-induced obesity. To elucidate the mechanisms involved in SKO-001 action, the effects of SKO-001 on adipose tissue were analyzed after 12 weeks of administration in HFD-induced obese mice. First, H&E staining of various fat depots revealed that SKO-001 supplementation reduced the average adipocyte size in all adipose tissues (SAT, VAT, and EAT) compared with those in the HFD group, suggesting that white adipocytes containing large lipid droplets were more likely to beige adipocytes upon SKO-001 administration (Fig. 4A, B). Correspondingly, the mRNA levels of *UCP-1*, a representative marker of beige adipocytes, were elevated by SKO-001 in all adipose tissues (SAT, VAT, and EAT) (Fig. 5A), as revealed by UCP-1 immunostaining (Fig. 5B). Second, changes in the mRNA expression of lipogenesis-related genes in adipose tissues were analyzed using RT-qPCR. As shown in Fig. 6A, the mRNA expression of adipogenic transcription factors, including *PPARγ* and *C/EBPα*, were significantly increased in the HFD group than in the ND group. SKO-001 treatment reduced *PPARγ and C/EBPα* levels in SAT. Furthermore, significantly lower mRNA levels of *SREBP-1c* were noticed in the SKO-001-treated groups. SKO-001 exhibited similar effects in VAT and EAT (Fig. 6B, C). Correspondingly, all protein levels were also reduced (Fig. 6A–C right panels), implying that SKO-001 had suppressive effects on HFD-induced lipogenesis in WATs, regardless of the source of adipose tissues.

### SKO-001 improves HFD-induced liver impairments

**Fig. 7:**
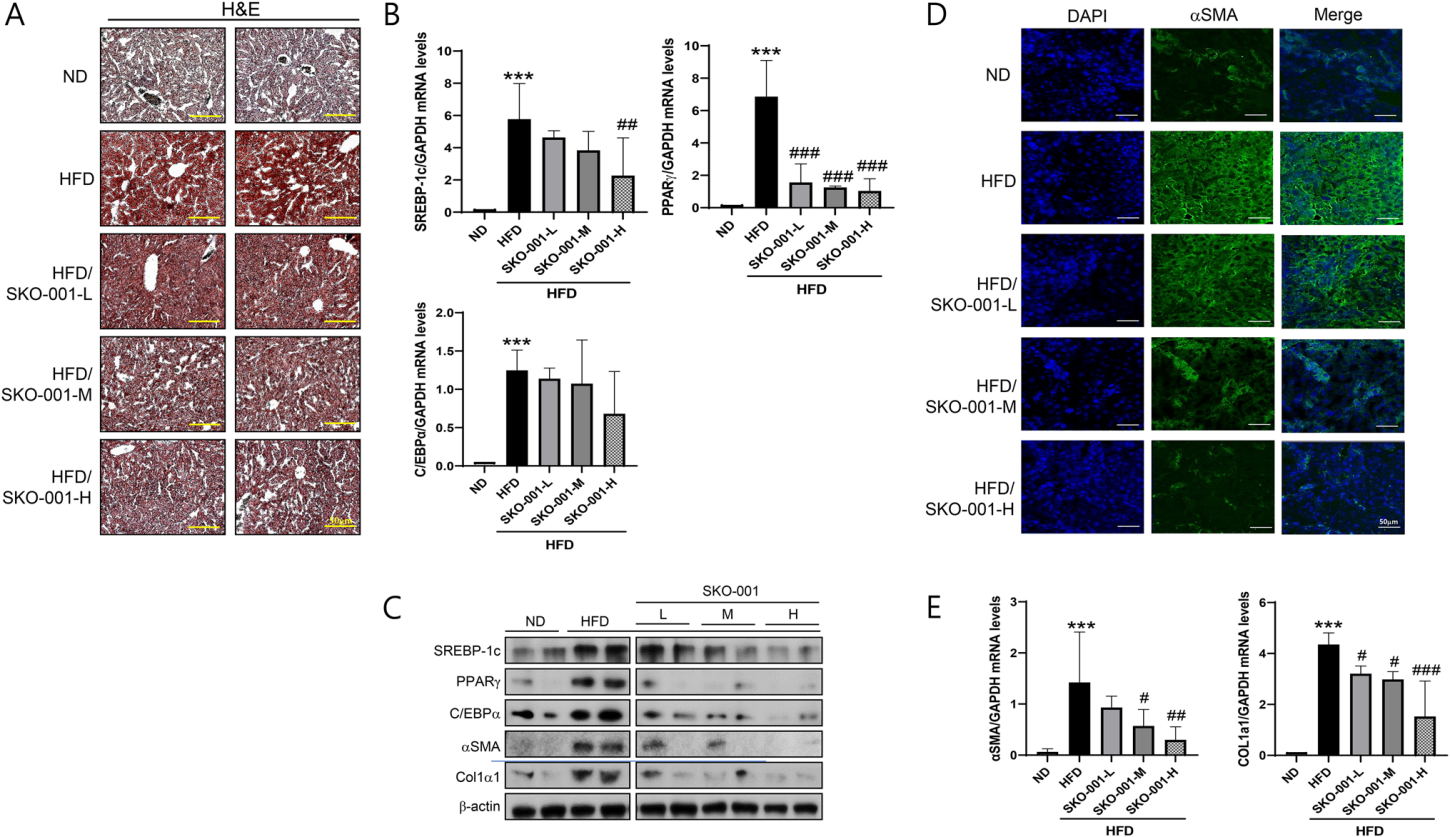
Effects of SKO-001 on liver tissues in HFD-induced obese mice. Liver tissues isolated from each group were subjected to Oil Red O staining (**A**) and α-SMA immunostaining (**D**) (Scale bar = 50 μm, 200 × magnification). The mRNA (**B**) and protein (**C**) levels of lipogenesis and fibrosis markers (**E**) were determined using real-time RT-qPCR (repeated three times, each in triplicate). Values are expressed as the mean ± SD. ***p < 0.001 *vs.* ND condition. ^#^*p* < 0.05, ^##^*p* < 0.01, ^###^*p* < 0.001 *vs*. HFD condition

Chronic obesity leads to accumulation of ectopic fat in tissues, notably in the liver. To further investigate the anti-lipogenic effects of SKO-001, hepatic lipid levels were measured using Oil Red O staining, and the results indicated that SKO-001 also reduced lipid accumulation in the liver (Fig. 7A). Likewise, the mRNA and protein levels of lipogenesis markers were also lower in the SKO-001-treated groups than in the HFD group, suggesting that SKO-001 improved the lipid metabolism profile in the liver, consistent with its effects on plasma determinants (Fig. 7B, C). Moreover, the mRNA levels of fibrosis markers, including *αSMA* and *Col1α1*, were decreased by SKO-001 (Fig. 7E), as revealed via immunostaining (Fig. 7D), which implies that SKO-001 may also have beneficial effects on liver fibrosis and cirrhosis.

## Discussion

Despite the drastic increase in the prevalence of obesity worldwide, effective therapeutic strategies to combat it are still lacking. Currently, several appetite suppressants are prescribed to treat severe obesity and related metabolic disorders [5]. Meanwhile, efforts are being made to overcome the adverse effects of existing drugs and expand the research on the discovery of novel therapeutic targets from natural sources. Some probiotics exert beneficial effects in reducing the body weight in rodents, and may possibly be used to treat human obesity [18, 27]. *Lactobacilli* and *Bifidobacteria* are the most well-studied probiotic strains that are effective against obesity and associated diseases [6].

In the present study, we discovered SKO-001, a probiotic strain isolated from *A. gigas,* as a possible anti-obesity agent and investigated its effects in HFD-induced obese mice. Based on the preliminary toxicity studies showing that any toxicological signs were not observed up to 6 × 10^10^ CFU/day (results not shown), three different doses of SKO-001 were selected and orally administered to mice along with the HFD for 12 weeks. The suppressive effects of SKO-001 on whole body weight appeared to be mild, and visible only at SKO-001-H (2 × 10^10^ CFU/day, at high dose), while its effects were limited to fat mass without any noticeable change in lean mass, suggesting that SKO-001 selectively works on fat mass reduction. In contrast, other obesity-induced parameters were significantly reversed by all doses of SKO-001, including SKO-001-L (low-dose). HFD-induced insulin resistance appears to be ameliorated by SKO-001, as evidenced by the increased plasma adiponectin levels and decreased leptin and insulin levels, in line with previous reports showing that *Lactobacillus gasseri* BNR17 induces weight reduction accompanied by the normalization of plasma levels of various cytokines [27].

Adipose tissue functions as an energy reservoir. However, obese adipose tissue fails to handle with excess fat, becoming dysfunctional. Notably, obesity-induced adipocyte hypertrophy triggers low-grade inflammation and subsequent insulin resistance [28]. Adipose browning, which refers to either the de novo differentiation of precursor cells to brown-like adipocytes or the trans-differentiation of white adipocytes to brown-like adipocytes, may offer a novel means of treating obesity and related metabolic diseases [10–12]. In the present study, SKO-001 administration resulted in reduced adipocyte size, along with increased levels of UCP-1, an important marker of browning, suggesting that SKO-001 may induce adipocyte browning, which may partly contribute to its anti-obesity effects by stimulating energy metabolism.

To further delineate the mechanism of action of SKO-001, we analyzed the expression of genes associated with lipogenesis in adipose tissue. The levels of SREBP-1c, which increases glycolysis and lipogenesis [17], were largely decreased by SKO-001 in a dose-dependent manner. PPARγ is a major transcription factor that modulates both glucose and lipid metabolism, and its expression in adipose tissue is associated with HFD-induced adipocyte hypertrophy and insulin resistance [29, 30]. SKO-001 treatment reverses the HFD-induced increase in *PPARγ* expression. Consistent with our findings, reduced *SREBP-1c* and *PPARγ2* expression were observed upon weight loss in women with obesity [31] and in HFD-induced obese mice [32]. In addition, mRNA expression of *C/EBPα*, a key regulator of adipogenesis and lipid accumulation [16], were completely suppressed by SKO-001, suggesting that inhibition of *SREBP-1c* and *C/EBPα* mRNA expression by SKO-001 resulted in reduced lipid accumulation. Interestingly, C/EBPα binds to the promoter and modulates the expression of leptin, which plays an important role in body weight homeostasis [33]. Consistently, reduced leptin levels were observed in the SKO-001-treated groups in our study, which may be linked to the anti-obesity effects of SKO-001. Similar effects of SKO-001 were observed in in vitro study that SKO-001 suppressed differentiation of 3T3-L1 preadipocytes, in parallel with reduced expression of lipogenesis genes (Suppl. Figure 5).

Increased fat accumulation in obesity is connected with dyslipidemia, collectively implying increased triglyceride, LDL-C, and TC levels, and decreased HDL-C levels and subsequently increasing the risk of coronary artery disease [4]. The results of the present study showed that SKO-001 improved HFD-induced changes in serum lipids by decreasing triglyceride, LDL-C, and TC levels, while concurrently increasing HDL-C levels. Furthermore, chronic obesity leads to the accumulation of ectopic fat in tissues, including the liver and muscles, resulting from the failure of the adipose tissue to handle the increased energy influx [13]. Indeed, SKO-001 significantly restored HFD-induced ectopic lipid accumulation in the liver, along with reduced mRNA expression of *SREBP-1c*, *C/EBPα*, and *PPARγ*. Additionally, SKO-001 reduced the mRNA levels of two important fibrosis markers, *α-SMA* and *Col1α1*, along with αSMA protein levels, as determined by α-SMA immunostaining, suggesting that SKO-001 may also be applicable to hepatic fibrosis and cirrhosis.

Long term diet containing high fat causes microbial dysbiosis, which can be ameliorated by probiotics including *L. plantarum*. For example, *L. plantarum* 1201 rebalanced the ratio of *firmicutes*/*bacteroidetes*, alleviating high-salt induced colitis [35]. Similarly, *L. plantarum* A29 increased percentage of *firmicutes phylum* compared with high-fat diet alone, which lowered body weights of HFD-induced obese mice [36]. Future studies to investigate the effects of SKO-001 on gut microbiota composition would be required. On the other hand, several metabolites produced by probiotics, such as lactic acid and short-chain fatty acids, also have shown beneficial effects against obesity and its related disorders [37]. It may be possible SKO-001 metabolites also contribute to the anti-obesity effects of SKO-001, which should be identified in future studies.

In summary, we demonstrated that SKO-001 suppresses HFD-induced body weight gain, improves serum biochemical parameters, and reduces ectopic lipid accumulation in the liver. The anti-obesity effects of SKO-001 may be due to the transcriptional downregulation of lipogenesis-related gene expression and induction of adipocyte browning. In addition, SKO-001 exerts beneficial effects on hepatic fibrosis. However, the detailed mechanisms of action of SKO-001 in adipose and liver tissues remain to be elucidated. It should also be clarified whether the anti-fibrotic effects of SKO-001 are independent of its effects on adipose tissue. Collectively, SKO-001 may be used as a novel therapeutic and/or dietary agent for ameliorating human obesity and its related metabolic disorders. Although the adverse effects of probiotics are rarely reported, the safety profile and human efficacy of SKO-001 should be investigated in future studies.

## Supplementary Information

Below is the link to the electronic supplementary material.

Supplementary file1 (PDF 1391 KB)

## Data Availability

All data generated in this study are contained within the article or supplementary material file.
