# Supplementary material for: Anti-obesity effects of Lactiplantibacillus plantarum SKO-001 in high-fat diet-induced obese mice
Source: Eur J Nutr. 2023 Feb 2;62(4):1611–22. doi: 10.1007/s00394-023-03096-x (PMC10195764; doi:10.1007/s00394-023-03096-x)
Supplement: Supplementary file 1 — Supplementary file1 (PDF 1391 KB) [file 394_2023_3096_MOESM1_ESM.pdf]

Anti-obesity effects of *Lactiplantibacillus plantarum* SKO-001 in high fat diet-induced obese mice

Mi Jin Choi[1], Hana Yu[1], Jea Il Kim[2], Hee Seo[3], Ju Gyeong Kim[3],
Seul-Ki Kim[3], Hak Sung Lee[4], Hyae Gyeong Cheon[1,2]

SAT

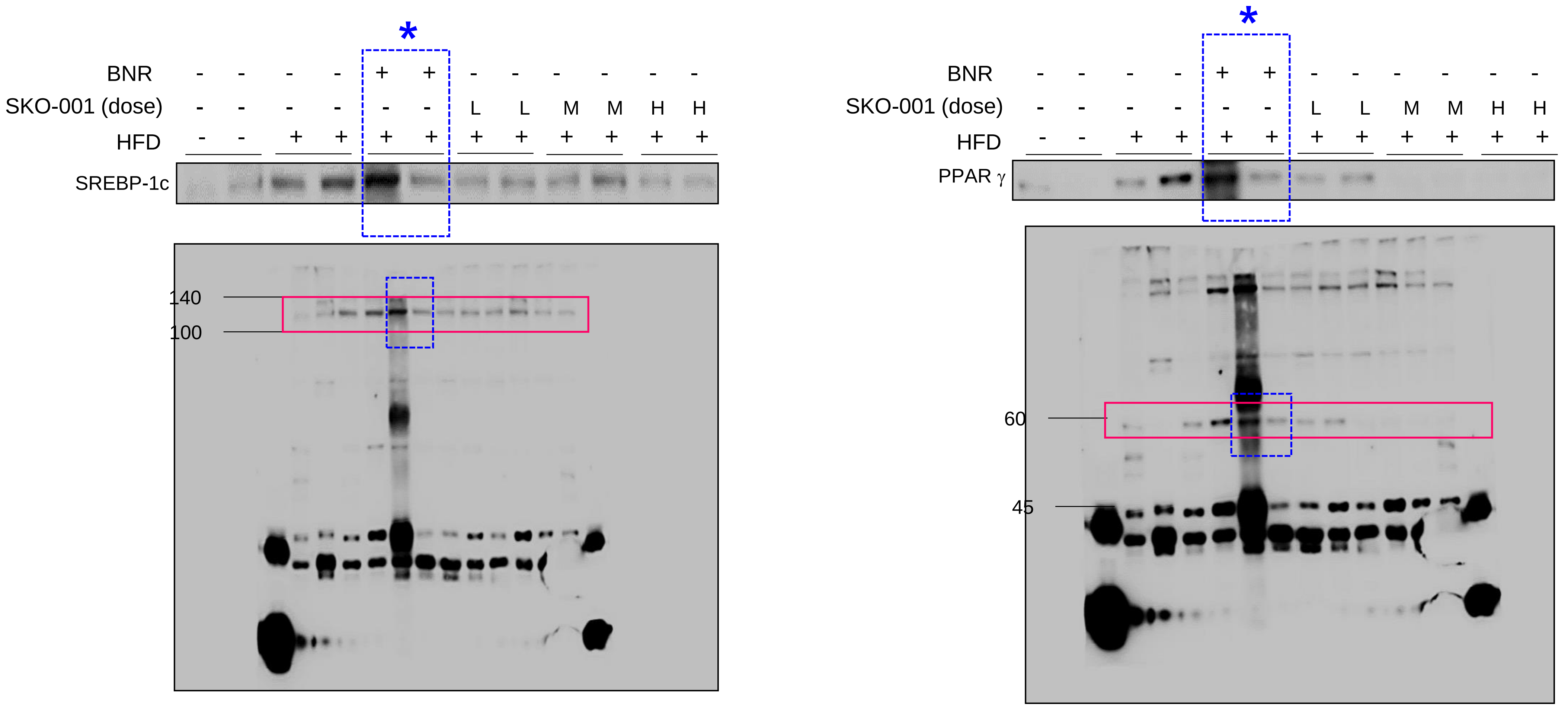

Supplementary Figure 1. Uncropped scans of western blot displayed in Fig. 6A

* BNR (lane 5-6) was not used in this manuscript, thus we cut out those lanes in figures

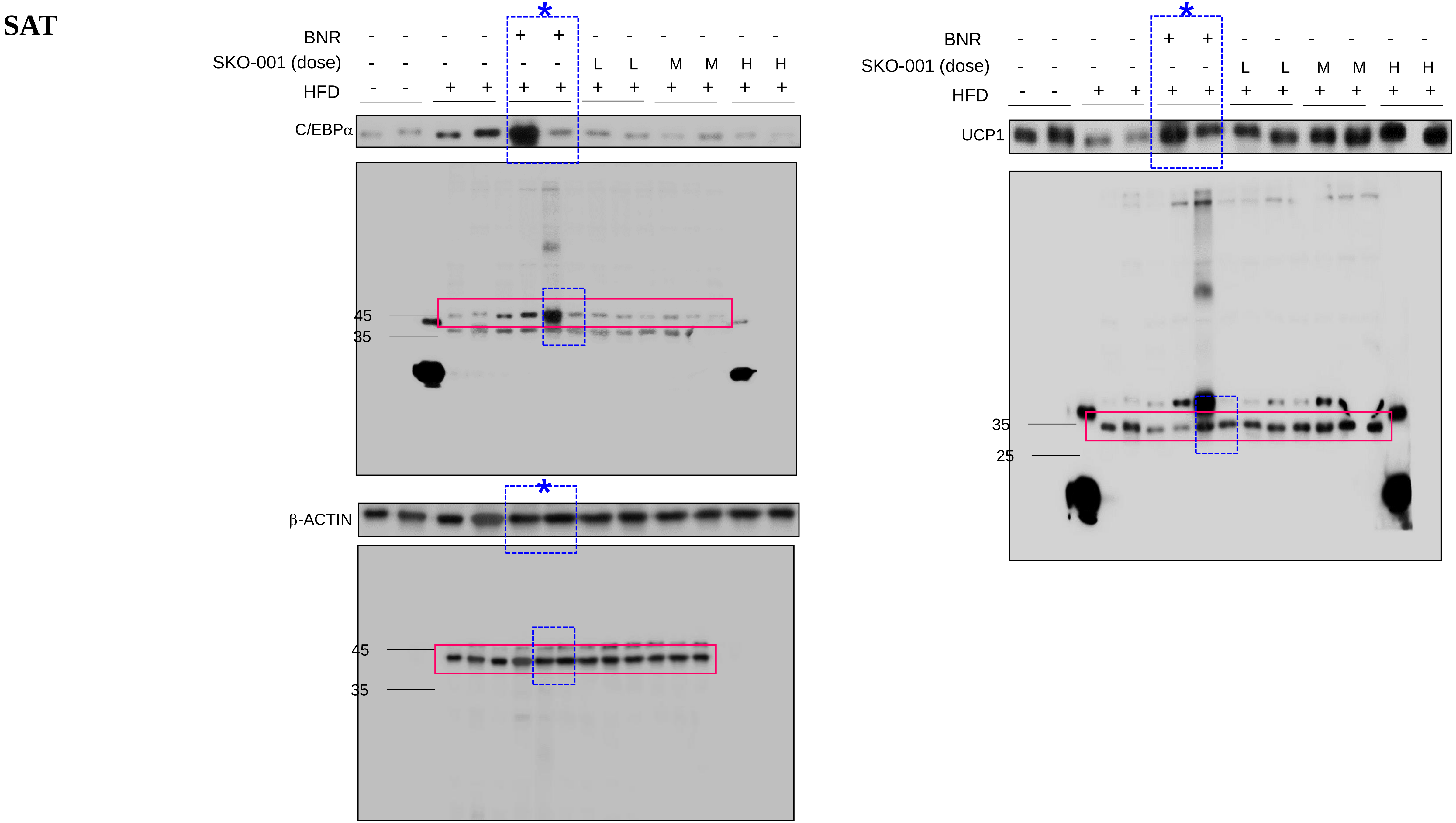

Supplementary Figure 1. Uncropped scans of western blot displayed in Fig. 6A

VAT

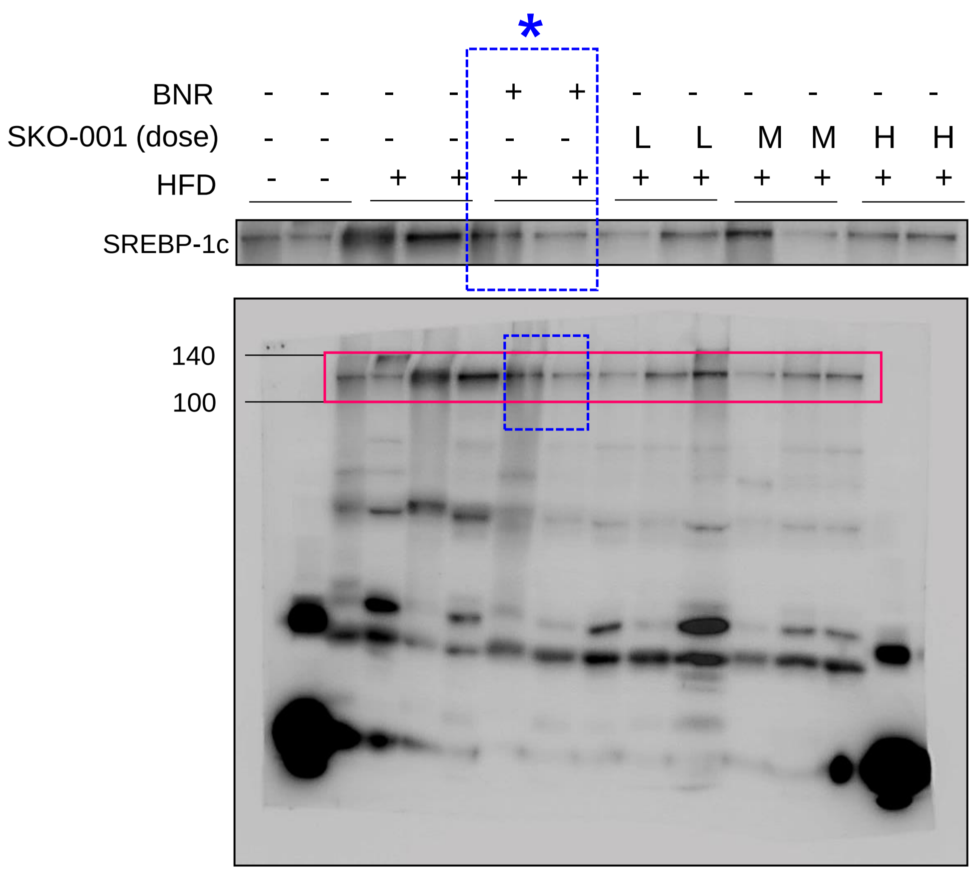

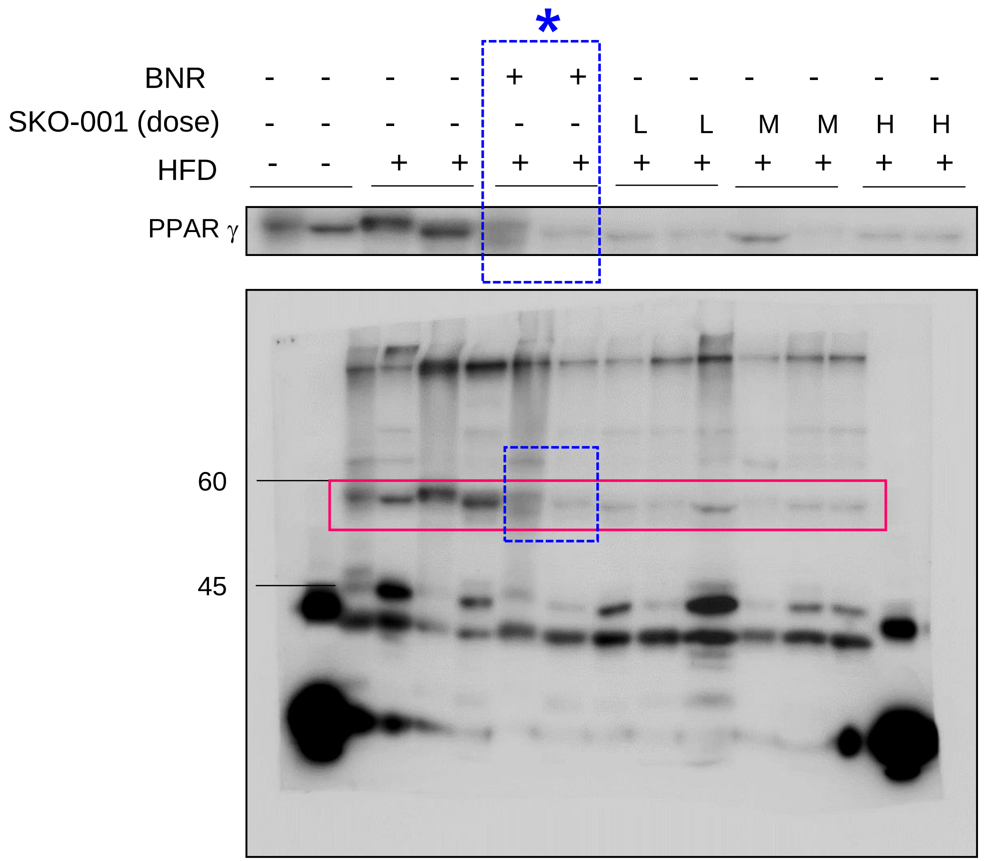

Supplementary Figure 2. Uncropped scans of western blot displayed in Fig. 6B

BNR (lane 5-6) was not used in this manuscript, thus we cut out those lanes in figures

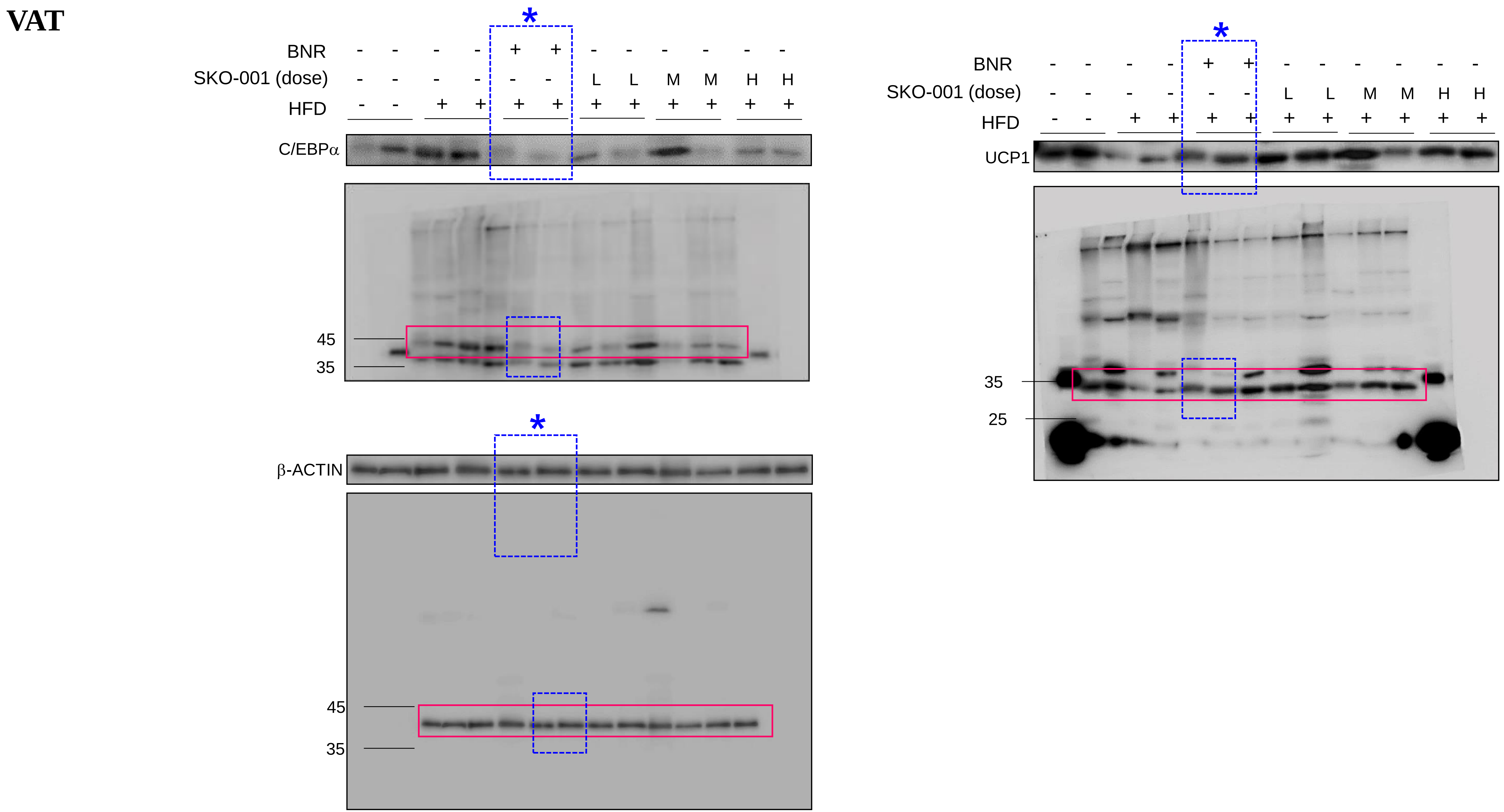

Supplementary Figure 2. Uncropped scans of western blot displayed in Fig. 6B

EAT

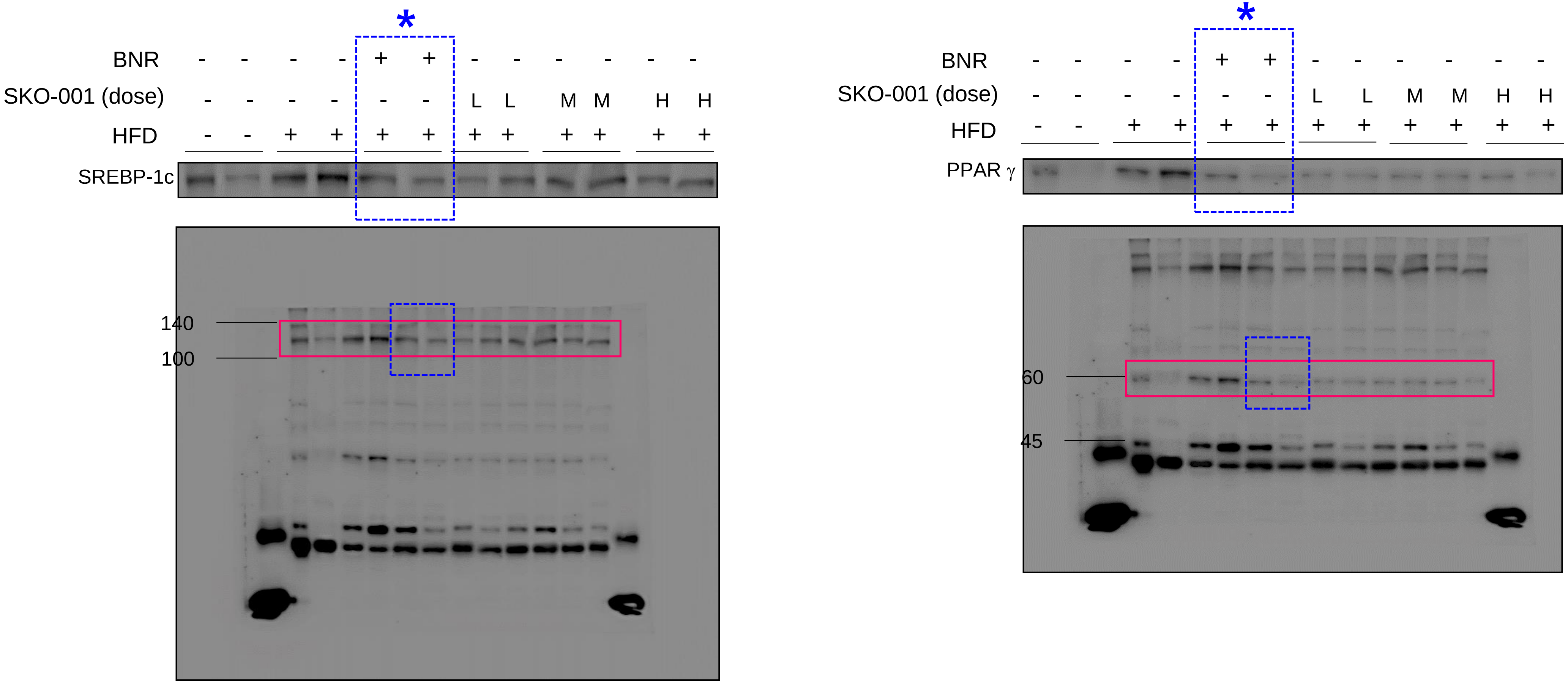

Supplementary Figure 3. Uncropped scans of western blot displayed in Fig. 6C

BNR (lane 5-6) was not used in this manuscript, thus we cut out those lanes in figures

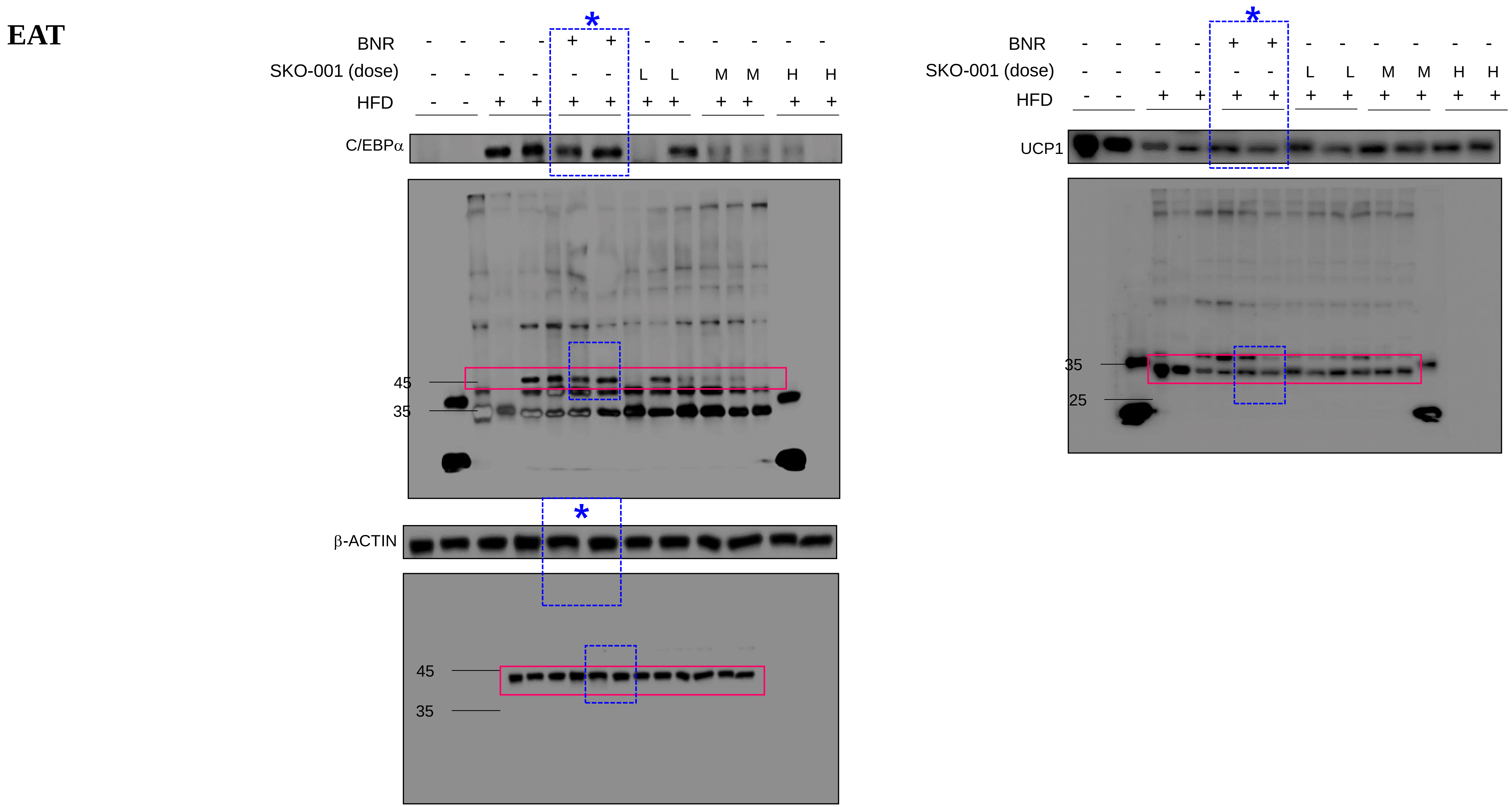

Supplementary Figure 3. Uncropped scans of western blot displayed in Fig. 6C

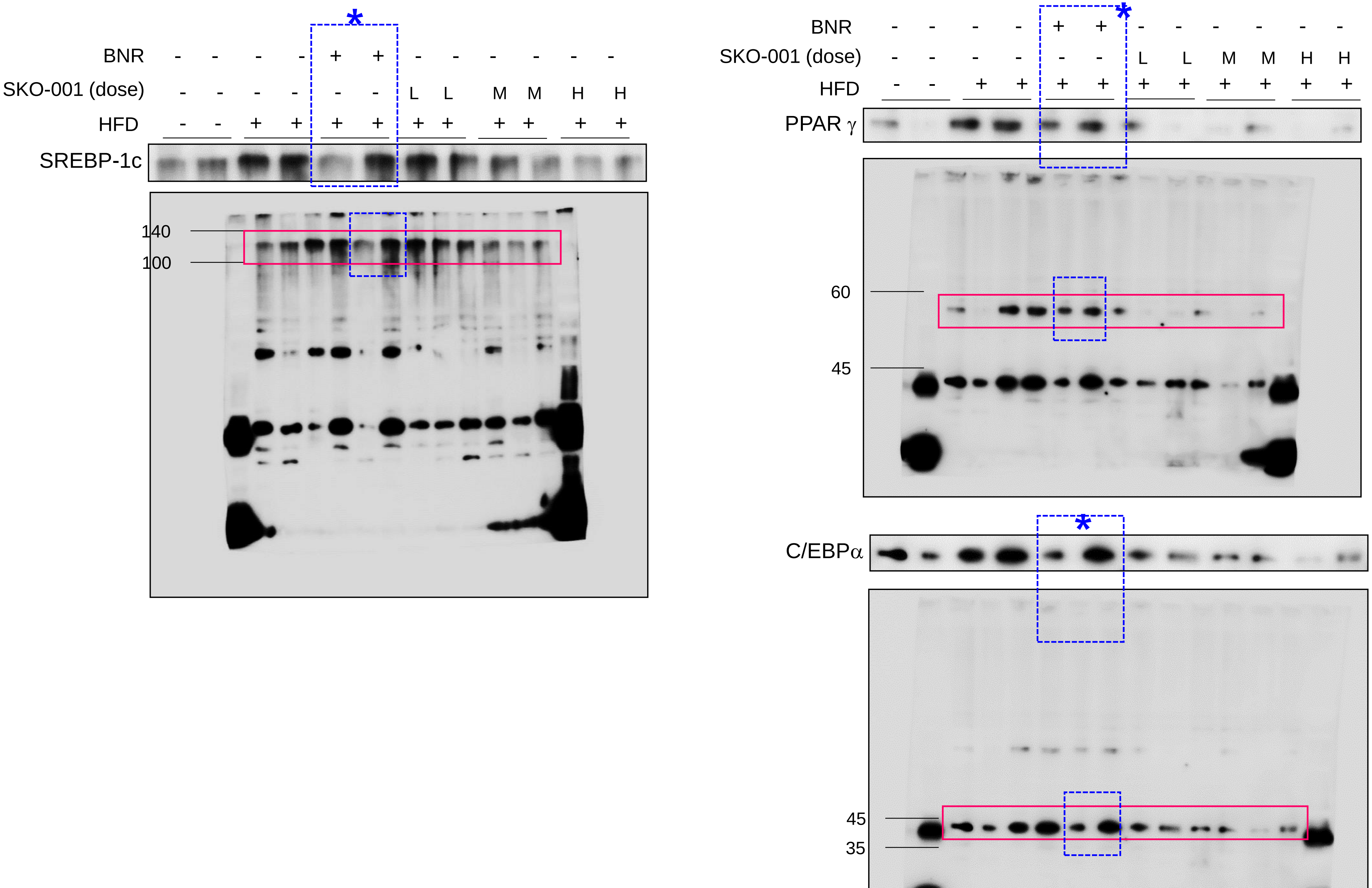

Supplementary Figure 4. Uncropped scans of western blot displayed in Fig. 7C

BNR (lane 5-6) was not used in this manuscript, thus we cut out those lanes in figures

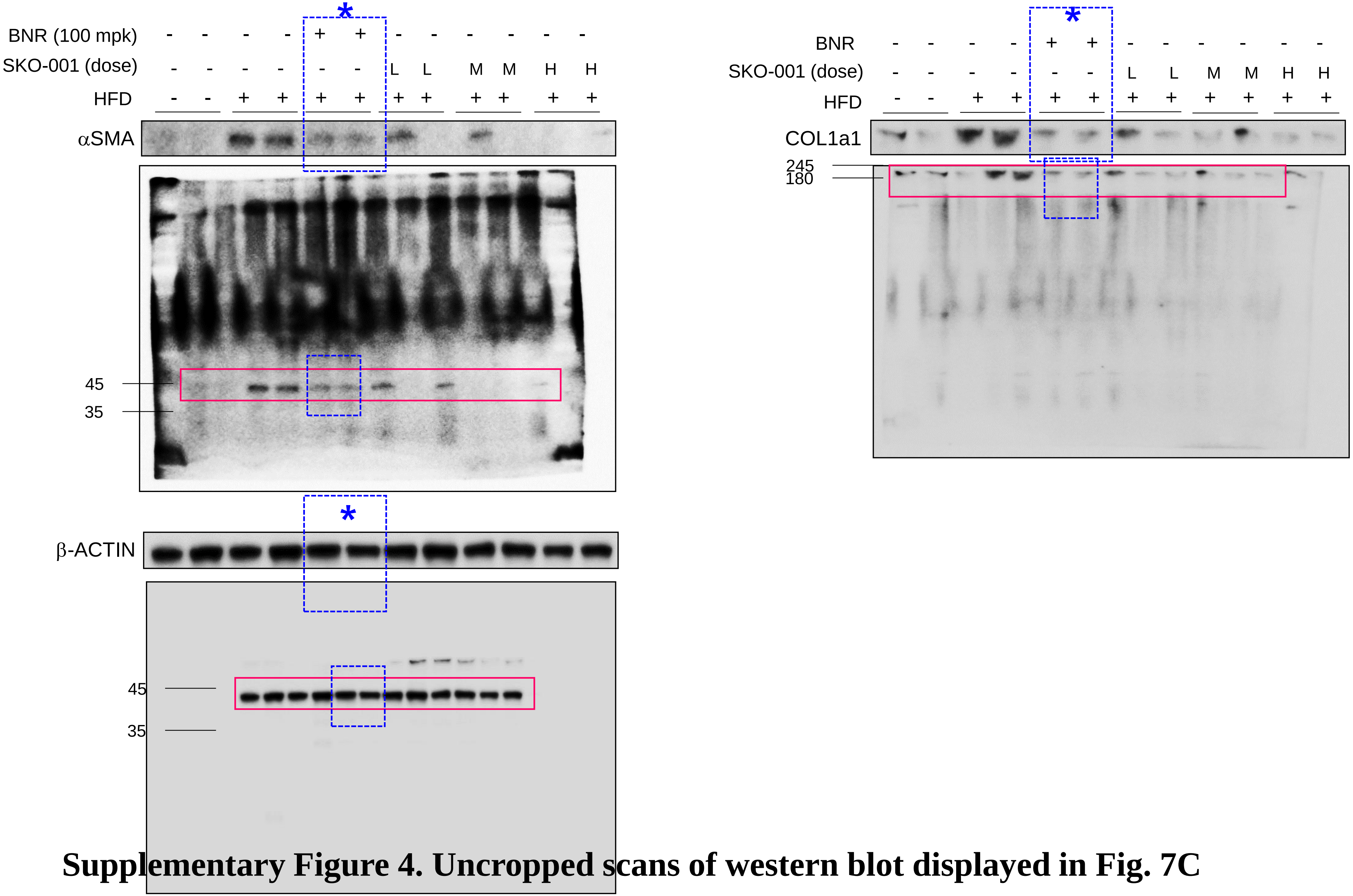

Supplementary Figure 4. Uncropped scans of western blot displayed in Fig. 7C

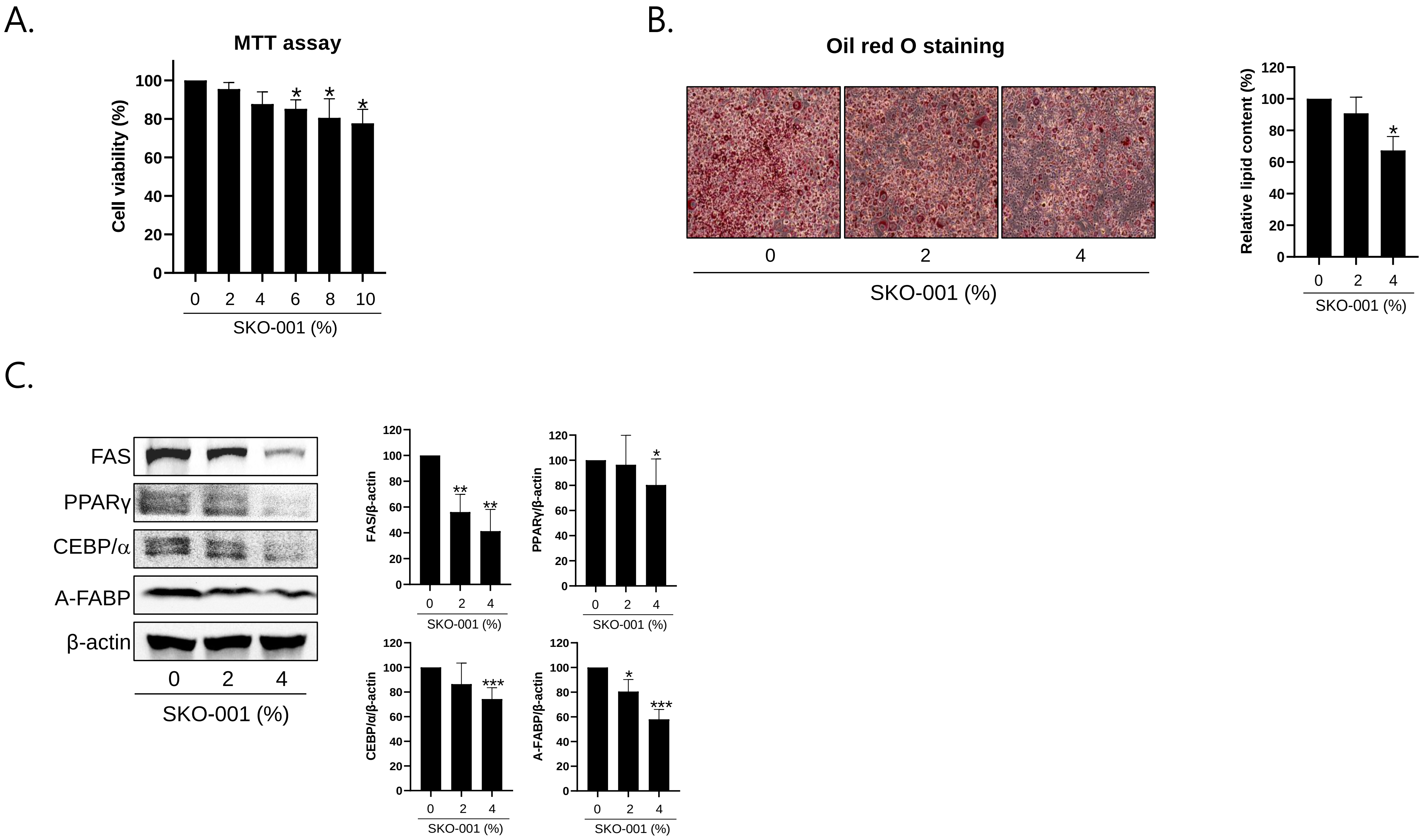

Supplementary Figure 5. Effects of SKO-001 on adipocyte differentiation
